# Highly Conserved Homotrimer Cavity Formed by the SARS-CoV-2 Spike Glycoprotein: A Novel Binding Site

**DOI:** 10.3390/jcm9051473

**Published:** 2020-05-14

**Authors:** Umesh Kalathiya, Monikaben Padariya, Marcos Mayordomo, Małgorzata Lisowska, Judith Nicholson, Ashita Singh, Maciej Baginski, Robin Fahraeus, Neil Carragher, Kathryn Ball, Juergen Haas, Alison Daniels, Ted R. Hupp, Javier Antonio Alfaro

**Affiliations:** 1 International Centre for Cancer Vaccine Science, University of Gdansk, Wita Stwosza 63, 80-308 Gdansk, Poland; monika.padariya@pg.edu.pl (M.P.); marcos1992@hotmail.com (M.M.); mlisowska@u.nus.edu (M.L.); ashita.nii@gmail.com (A.S.); robin.fahraeus@inserm.fr (R.F.); 2 Sharp Life Science (EU) Limited, Oxford Science Park, Edmund Halley Rd, Oxford OX4 4GB, UK; judith.nicholson@sharp-ls.eu; 3 Department of Pharmaceutical Technology and Biochemistry, Faculty of Chemistry, Gdansk University of Technology, Narutowicza St 11/12, 80-233 Gdansk, Poland; maciej.baginski@pg.edu.pl; 4 Institute of Genetics and Molecular Medicine, University of Edinburgh, Edinburgh, Scotland EH4 2XR, UK; n.carragher@ed.ac.uk (N.C.); kathryn.ball@ed.ac.uk (K.B.); 5 Department of Infectious Disease, Edinburgh, Scotland EH4 2XR, UK; juergen.haas@ed.ac.uk (J.H.); A.Daniels@sms.ed.ac.uk (A.D.)

**Keywords:** SARS-CoV-2, coronavirus disease 2019 (COVID-19), spike glycoprotein, variability, molecular docking, molecular dynamics, inhibitors, trimer cavity, binding site

## Abstract

An important stage in severe acute respiratory syndrome coronavirus 2 (SARS-CoV-2) life cycle is the binding of the spike (S) protein to the angiotensin converting enzyme-2 (ACE2) host cell receptor. Therefore, to explore conserved features in spike protein dynamics and to identify potentially novel regions for drugging, we measured spike protein variability derived from 791 viral genomes and studied its properties by molecular dynamics (MD) simulation. The findings indicated that S2 subunit (heptad-repeat 1 (HR1), central helix (CH), and connector domain (CD) domains) showed low variability, low fluctuations in MD, and displayed a trimer cavity. By contrast, the receptor binding domain (RBD) domain, which is typically targeted in drug discovery programs, exhibits more sequence variability and flexibility. Interpretations from MD simulations suggest that the monomer form of spike protein is in constant motion showing transitions between an “up” and “down” state. In addition, the trimer cavity may function as a “bouncing spring” that may facilitate the homotrimer spike protein interactions with the ACE2 receptor. The feasibility of the trimer cavity as a potential drug target was examined by structure based virtual screening. Several hits were identified that have already been validated or suggested to inhibit the SARS-CoV-2 virus in published cell models. In particular, the data suggest an action mechanism for molecules including Chitosan and macrolides such as the mTOR (mammalian target of Rapamycin) pathway inhibitor Rapamycin. These findings identify a novel small molecule binding-site formed by the spike protein oligomer, that might assist in future drug discovery programs aimed at targeting the coronavirus (CoV) family of viruses.

## 1. Introduction

The global pandemic developing from December 2019 by a strain of severe acute respiratory syndrome coronavirus 2 (SARS-CoV-2) can cause coronavirus disease 2019 (COVID-19) disease. This emergent variant adds to the additional coronavirus strains that can infect humans including human coronavirus OC43 (HCoV-OC43), human coronavirus HKU1 (HCoV-HKU1), SARS-CoV, human coronavirus 229E (HCoV-229E), human coronavirus NL63 (HCoV-NL63), and human coronavirus NL63 (HCoV-NL63) [1,2,3,4,5,6]. Coronaviruses (CoVs) are positive-sense, enveloped, single-stranded RNA viruses that are classified taxonomically as a family *Coronavirdiae* and order *Nidovirales* [4]. There are four genera of CoVs, including αCoV, βCoV, δCoV, and γCoV; most δCoVs and γCoVs target avians, whilst αCoVs and βCoVs infect rodents and bats [1,7,8]. Severe acute respiratory syndrome CoV (SARS-CoV) outbreaks have also emerged previously creating an epidemic [2,4,9,10,11,12,13]. Although the mortality of MERS-CoV, SARS-CoV, and SARS-CoV-2 is substantial, there are no preventative vaccines or drugs available to treat patients infected with the virus [9,11,12]. The current public health emergency of international concern (PHEIC) by the World Health Organization (WHO) has declared SARS-CoV-2 (COVID-19; a novel βCoV) as a pandemic threat. The data obtained from WHO (08/May/2020) suggest that the virus has caused 3,759,967 infections, 259,474 deaths, and it has affected over 200 countries.

The Open Reading Frame 1ab (ORF1ab) of SARS-CoV-2 encodes for three proteins that are broadly recognized as drug targets, since they are key components for infections and disease progression: the SARS-CoV-2 protease [14,15], the RNA-dependent RNA Polymerase (RdRP) [14,16,17], and the SARS-CoV-2 spike (S) glycoprotein [15,18,19,20]. The SARS-CoV-2 protease processes the polyproteins that are translated from the viral RNA, and it has been heavily studied using small molecules inhibitors [15]. To penetrate the host, the SARS-CoV-2 makes use of homotrimeric class I glycosylated fusion spike protein [18,21,22]. Fusion of the viral and host cell membranes is facilitated by the spike glycoprotein, which undergoes a significant conformational change upon fusion [18,21,22].

SARS-CoV-2 studies suggest [18,23,24] that the spike glycoprotein functions as a homotrimer. The recognition and subsequent fusion of the viral and cellular membranes are triggered by the S1 subunit of the spike protein, which binds the host cell receptor; angiotensin converting enzyme-2 (ACE2) [16,25,26,27,28,29,30,31]. Several insights from structural biology are consistent with the role for this domain in affecting the infection rate of the virus. This host–virus interaction is mediated by the receptor binding domain (RBD) domain from S1 subunit of SARS-CoV-2 spike glycoprotein that forms a hinge-like conformation [18,32], i.e., “down” and “up” states that represents the host cell receptor-inaccessible and receptor-accessible [18]. This receptor-accessible “up” conformation exists in a highly fluctuating state [33,34,35,36]. Binding to the host target destabilizes the pre-fusion homotrimer, which sheds off the S1 subunit, and allows for the transition of the S2 subunit to a highly stable postfusion conformation [18]. Interestingly, protein-mediated cell–cell fusion assays suggest that SARS-CoV-2 spike protein displays an elevated plasma membrane fusion capacity when compared to that of SARS-CoV [32,37].

Several studies have aimed to define the mechanism of binding of SARS-CoV-2 to the host cell receptor [38]. Molecular dynamics simulations of the spike (RBD)-ACE2 complex, over 10 ns indicated that spike(RBD)-ACE2 binding free energy for SARS-CoV-2 is better than for the SARS-CoV [39]. Similarly, other studies have shown that the SARS-CoV-2 spike protein has a better binding affinity to ACE2 at two different “up” angles of the RBD domain than the SARS-CoV [40]. Structural features at the spike-ACE2 interface suggest that residues Q493 and P499 from the spike RBD domain are responsible for maintaining protein–protein stability [41]. Using a virtual high-throughput screening approach, small-molecules have been identified that can interact with the RBD domain of SARS-CoV-2 spike protein [42]. Natural compounds present in *Curcuma* sp., *Citrus* sp., *Alpiniagalanga*, and *Caesalpiniasappan* could also target the RBD domain of the SARS-CoV-2 spike glycoprotein, the protease domain (PD) from ACE2, and the SARS-CoV-2 protease [20]. A set of B cell and T cell epitopes derived from the spike and nucleocapsid proteins that map identically to SARS-CoV-2 proteins, were identified as potential vaccine candidates [23].

Applying an integrative, antiviral drug repurposing methodology, the interplay between the CoV–host interactome and drug targets in the human protein–protein interaction network have been defined [43]. Bioinformatics methodologies were used to identify neutralizing antibodies that might interact with interfaces formed by the spike glycoprotein and the ACE2 host cell receptor [24]. By targeting the RBD domain of the spike protein using docking experiments, Kanishka et al. identified small molecule inhibitors [44]. In the majority of studies, the most common strategy is focused on targeting the interface formed by SARS-CoV-2 spike glycoprotein and the ACE2 host cell receptor (i.e., spike(RBD)-ACE2). Currently, there are no robust drugs for wide-spread dissemination available against coronaviruses including; the SARS-CoV-2 virus. Due to the relatively rapid spread in the current outbreak and the relatively high mortality rate (3.5%), more rapid development of new or repurposed antiviral drugs is of high value. Although the majority of drug discovery programs target classically druggable enzymes encoded by the virus, such as the viral RNA polymerase inhibited by remdesivir [13,17,45], there is a paucity of information concerning the other regions of spike glycoprotein outwith the ACE2-binding domains, especially the domains interacting with the viral membrane.

**Figure 1:**
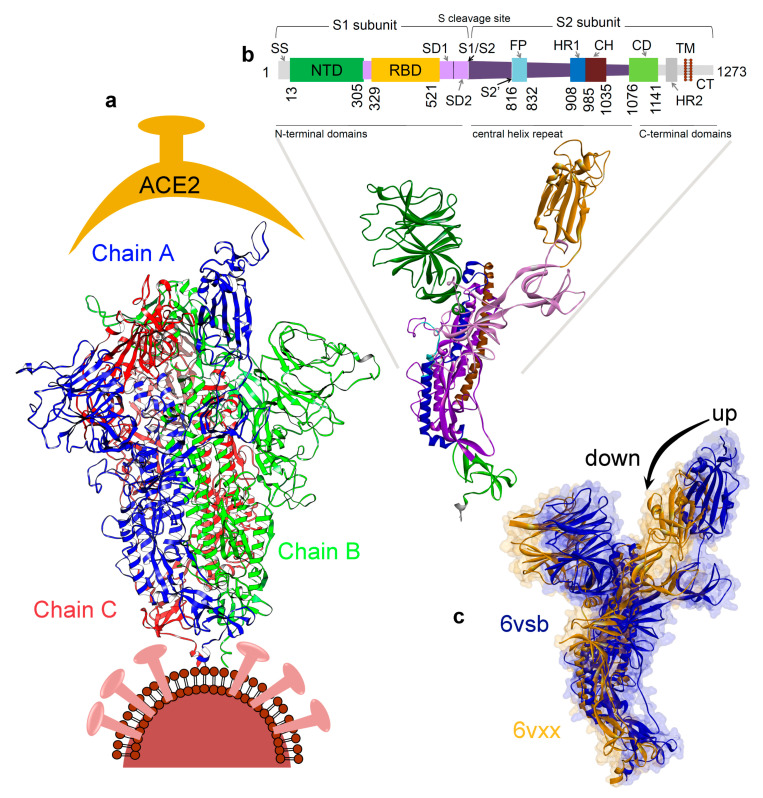
Severe acute respiratory syndrome coronavirus 2 (SARS-CoV-2) spike protein structure and function. (**a**) The homotrimer spike glycoprotein (PDB ID. 6vsb) [18]. (**b**) Different domains of the spike protein that includes; signal sequence (SS), the N-terminal domain (NTD), receptor-binding domain (RBD), subdomain 1 and 2 (SD1&2), protease cleavage sites (S1/S2/S2′), fusion peptide (FP), heptad repeat 1 and 2 (HR1&2), central helix (CH), connector domain (CD), transmembrane domain (TM), and cytoplasmic tail (CT) [18,19]. (**c**) Receptor binding domain (RBD) illustrating the “up” or “open” (PDB ID. 6vsb [18]) and “down” or “closed” (PDB ID. 6vxx [31]) conformation.

The SARS-CoV-2 spike protein is a homotrimer composed of three monomers (chains A, B, and C; Figure 1a). Each monomeric protein contains an N-terminal ACE2 binding domain (receptor binding domain; RBD), a central helix/heptad repeat, and a C-terminal region that interacts with the plasma membrane [18]. Homotrimer spike protein assembly from monomeric forms can be rate limiting in cells, suggesting a possible space for intervention on the viral life cycle [46]. Our current study focuses on understanding the variability of the trimer spike glycoprotein in SARS-CoV-2 with respect to the genomes from other coronavirus strains, and identifying the changes in the molecular properties due to conformational flexibility in the spike protein. The analysis suggests that residues in the S2 subunit are less variable compared to the other regions. In addition, the molecular dynamics simulations (MDS) identified that residues from the RBD domain obtained substantial flexibility which may be an obstacle in finding active hits. By contrast, residues in the S2 subunit (trimer cavity) showed the least flexibility representing a novel binding region for ligands. This information was used to identify potentially novel drug pockets or the active site regions specifically in the oligomeric SARS-CoV-2 spike glycoprotein.

We performed MD simulations on the monomeric and trimeric form of the SARS-CoV-2 spike glycoprotein, and developed a virtual screening using a Food and Drug Administration (FDA) approved chemical library. We identified and focused on an apparent cavity formed by three subunits (the homotrimer), that our simulations suggest can mediate dynamic movements that mimic a “bouncing spring” or a “sarrus linkage (converting a circular motion to a linear motion or vice versa)” when interacting with the ACE2 host cell receptor. This motion might be important in the fusion of the virion and the host cell membrane. We hypothesized that such a cavity formed by three monomers or subunits of the spike protein (i.e., chains A, B, and C) might form an acceptor for small molecules, and we asked whether small molecules could be identified with a relatively high binding energy. We identified several known compounds with predicted binding energy of GBVI/WSA dG (Generalized-Born Volume Integral/Weighted Surface area) from −35 to −71 kcal/mol, some of which are already proposed for clinical trials including an mTOR (mammalian target of Rapamycin) pathway inhibitor, Sirolimus (Rapamycin; a macrolide type; NCT03901001 not yet recruiting) [47,48,49] and Ritonavir (open-label trial in hospitalized adults with severe COVID-19) [48,50,51,52]. A recent study that screened hundreds of approved molecules in a SARS-CoV-2 assay using artificial intelligence-enabled phenomic assays [53], also identified Sirolimus (Rapamycin) as a promising candidate. In addition to the macrolides, one of the top hits we have identified, Chitosan, has a recently reported derivative inhibiting SARS-CoV-2 coronavirus replication in cell lines [54,55]. A previous study has also shown that the chitosan derivatives can interact with the spike protein and block its interaction with the host receptor [56]. Our data suggest a mechanism whereby Chitosan (and possibly its derivatives), as well as macrolide type molecules, might bind to a pocket formed by the spike protein trimer and provide a novel domain to focus on for future drug discovery projects.

## 2. Material and Methods

### 2.1. Bioinformatics Analysis of the SARS-CoV-2 Spike Variability

A total of 791 viral genome sequences were downloaded from the Global Initiative on Sharing All Influenza Data platform (GISAID) [57], in order to define the evolutionary variability in different domains of the spike glycoprotein. Only genomes with high coverage and complete sequences were selected. Further filtering was applied to obtain complete sequences on the targeted domains which reduced the total number of strains to 768. Total protein sequences were acquired from 3 frame translation using the transeq tool from EMBOSS (European Molecular Biology Open Software Suite) package (version 6.5.7) (European Bioinformatics Institute (EMBL-EBI), Hinxton, Cambridge, UK) [58]. The amino acid chains from the spike glycoprotein were aligned to the reference protein (PDB ID. 6vsb [18,59]) using MUSCLE [60]. Variations in the amino acid or the residue changes were scanned on the entire spike protein sequence, along with two areas of interest in the multiple alignment file, focusing on a subset of the S2 subunit (HR1, CH, and CD domains) and the RBD domain (Figure 1 and Tables S1–S3).

### 2.2. Structural Bioinformatics, Molecular Dynamics

The cryo-EM (cryogenic electron microscopy) homotrimer structure of SARS-CoV-2 spike glycoprotein was retrieved from the Protein Data Bank database (http://www.rcsb.org/pdb; PDB ID. 6vsb; Figure 1) [18,59]. In addition, the missing amino acid (residues range: 67–78, 96–98, 143–155, 177–186, 247–260, 329–334, 444–448, 455–490, 501–502, 621–639, 673–686, 812–814, and 829–852) coordinates in the structure of SARS-CoV-2 spike glycoprotein were built using the swissmodel (Figure 1) [61]. Molecular dynamics simulations were carried on the model systems as per the standardized pipelines [62,63,64] (detailed method explained in the supplementary materials; File S1). The GROMACS 4.6.5 [65] program (GROMACS; Groningen Machine for Chemical Simulations, University of Groningen, Groningen, The Netherlands) was used to perform MD calculations assigning the CHARMM27 forcefield [66]. We performed 100 ns molecular dynamics simulations on two systems: (i) The monomeric form and (ii) the homotrimer form of the spike protein. In our analysis of the MD simulations, the dynamics of the monomeric form of the spike protein serves as control to the homotrimer, which is the functional unit. Initially, the model systems were energy minimized, which provides a base-line model structure and resolves poorly-resolved conformations often found in crystal structures [13,18,62,67,68,69,70]. A simulation box of solvent atoms is then added to enhance simulation realism. Following that, using the NPT (number of particles (N), system pressure (P), and temperature (T); isobaric-isothermal) thermodynamic ensemble, equilibration of the systems was performed to adjust solvent molecules with counter ions in the simulation box [71]. These equilibrated systems were subsequently used to perform the final MD production runs for 100 ns, and results were analyzed using GROMACS [56], BIOVIA Discovery Studio (DassaultSystèmes, BIOVIA Corp., San Diego, CA, USA), Chimera, and visual molecular dynamics (VMD) tools [71,72,73].

### 2.3. In Silico Structure Based Virtual Screening

Structure-based virtual screening (SBVS) is an application of in silico methods that identify promising lead molecules from chemical libraries or databases. These methods are computational counterparts of experimental biological evaluation methods, such as high-throughput screening (HTS). FDA approved drug libraries were retrieved from Target Molecule Corp. (TargetMol; www.targetmol.com) and Selleck Chemicals (Selleckchem; www.selleckchem.com) vendors. The SBVS against the SARS-CoV-2 spike glycoprotein was performed using the Molecular Operating Environment (MOE; Chemical Computing Group Inc., Montreal, QC, Canada) package [74,75]. Receptor–ligand binding or docking using the CHARMM27 forcefield [66] was evaluated using the GBVI/WSA ∆G scoring function [76]. The compounds showing best energies with the spike protein were selected for further analysis. GBVI/WSA ∆G is a forcefield based scoring function which determines the free energy of binding of the ligand from a given position [75]. In addition, we have also selected the compounds that showed comparatively stable interactions with the homotrimer spike protein. Applying the “Triangle Matcher”placement method, receptor–ligand docking was performed defining the receptor as rigid and ligands as flexible [74,75].

## 3. Results

### 3.1. Investigating the SARS-CoV-2 Spike Glycoprotein Reveals a Cavity with Potential Utility as a Drug-Binding Pocket

#### 3.1.1. Variability in the Spike Glycoprotein

We were interested to define the evolutionary variance in the SARS-COV-2 spike protein. Understanding regions of high and low variance can identify domains that may be functionally conserved and potentially important to the virus life cycle, or those under positive evolutionary pressure whose selection might avoid the immune system.

**Figure 2:**
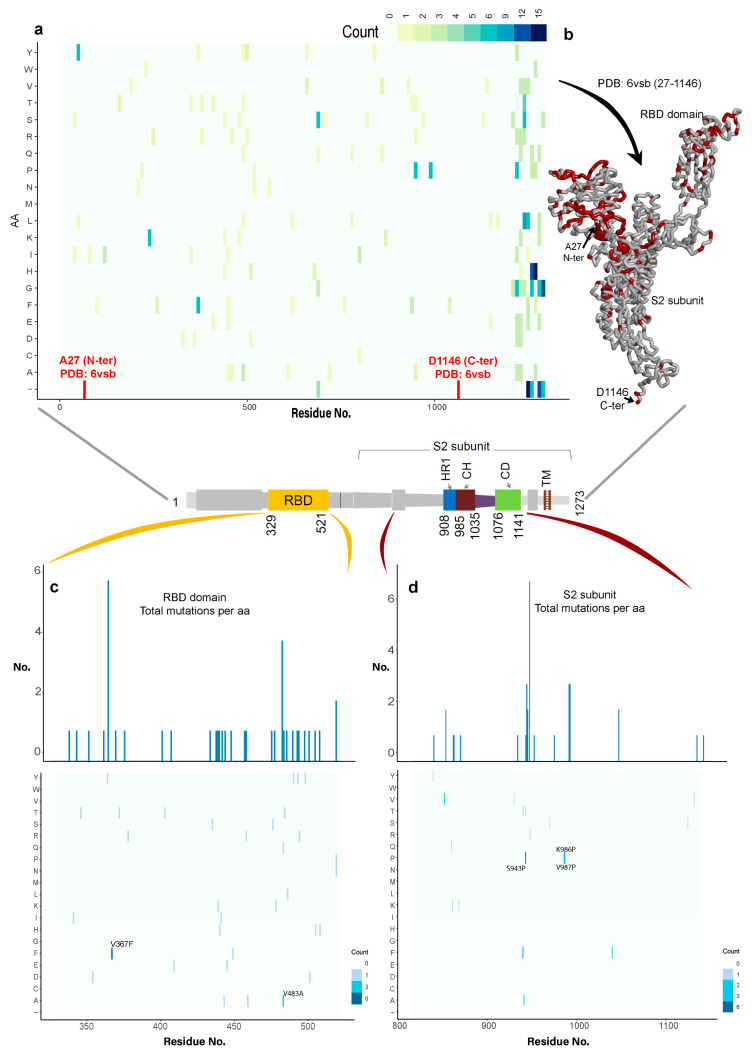
Variability in the SARS-CoV-2 spike protein. (**a**) The heat map contains the amino acid substitutions for each position in the spike protein compared to the consensus sequence (wild-type) from the alignment of 791 SARS-CoV-2 strains from the Global Initiative on Sharing All Influenza Data (GISAID) database [57]. (**b**) Represents the previous variations over the spike protein structure, marking in red color the spots of variability. (**c**,**d**) Analysis of the amino acid substitutions in the receptor binding domain (RBD) domain and in the S2 subunit (HR1, CH, and CD domains), including a bar plot with the total number of changes for each position.

Examining the variability of the spike protein in SARS-CoV-2 and its different domains, a total of 791 genome viral sequences were retrieved from GISAID [57]. A global view of the mutation space of the virus is presented in Figure 2a, which represents the amino acid substitutions in bins of 10 aminoacids across the spike glycoprotein. These hotspots of variation are mostly confined to the NTD and the RBD domains (Figure 1b and Figure 2b). We investigated the variability in the entire sequence of the spike protein, focusing on the regions that showed low-variability in the structure (Figure 2).

By investigating the variations in the residue changes across the entire spike protein sequence or all the regions of lower variability (Figure 2a and Table S1), the S2 subunit exhibited the lowest sequence variability (residue range: 816–1141; Figure 1b and Figure 2c). Moreover, previous studies have identified that the active site region for this spike protein is located in the RBD domain which interacts with the ACE2 host cell receptor [18,20,26,38,39,40,42,44]. Comparing the variability of the RBD domain and S2 subunit domains, the RBD domain was shown to contain more mutations in its region compared to the S2 subunit (HR1, CH, and CD) domains (Figure 2b,c, and Tables S1–S4). These data suggest that during mutation by natural selection, the viral-host “arms race” might operate more frequently on the RBD domain. By contrast, the S2 subunit conservation is suggestive of an important core function where mutations cannot be tolerated. These findings prompted our focus on the S2 subunit as an important region to investigate for identifying potentially novel druggable pockets.

#### 3.1.2. Molecular Properties and the Dynamics of the Spike Protein

**Figure 3:**
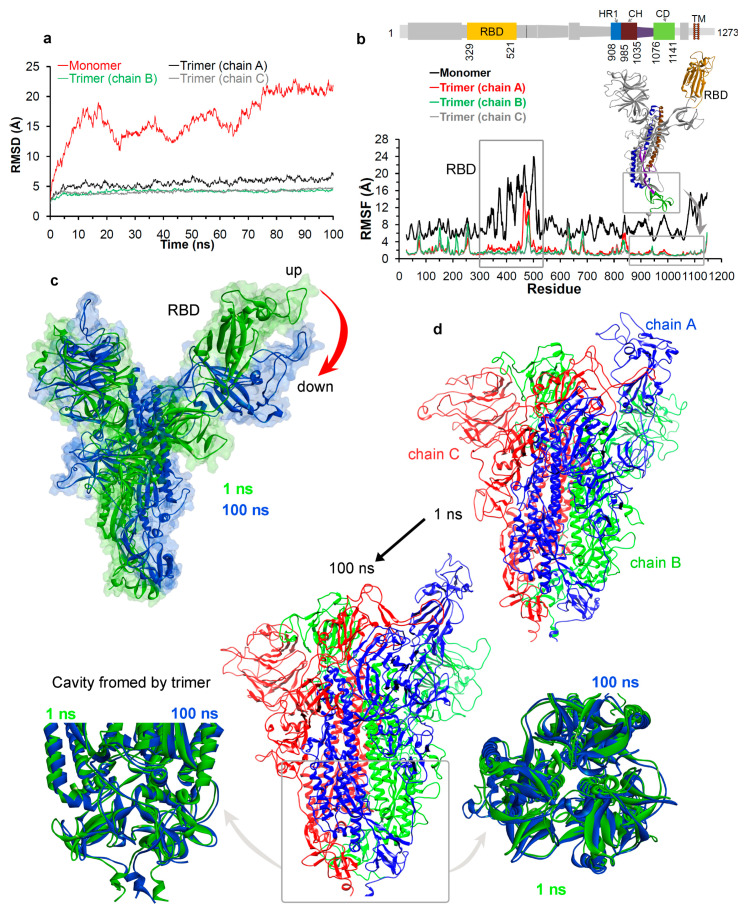
Conformational dynamics of the SARS-CoV-2 spike glycoprotein. (**a**,**b**) RMSD and RMSF of the monomeric and trimeric forms. (**c**) The “up” and “down” state traced during the MD simulations of the monomeric form. (**d**) The conformation dynamics of homotrimer spike protein, as well as the highlighted cavity formed by the trimer state and its evolution over 100 ns of the MD simulation. RMSD, root mean square deviation; RMSF, root mean square fluctuations; MD, molecular dynamics.

We next traced the dynamics of different domains in the spike protein using MD simulations (Figure 1b). The simulated model systems of the spike protein in the monomer and the homotrimer forms were first processed to check the stability of the protein. Stability of the simulated spike protein in both forms in the solvent environment was traced by RMSDs (root mean square deviation), a time dependent change in the non-hydrogen atoms (Figure 3a). The RMSD plots (Figure 3a) suggest that the trimer form of the spike protein is more stable compared to the monomer form. In addition, chain A in trimer has a higher RMSD (~2 Å) compared to the other two chains which is a consequence of the fact that the “up” (or ACE2-active) conformation [18] induces flexibility. Since the monomer form has a higher RMSDs compared to the trimer (Figure 3a), we performed independent triplicates (MDS was repeated three times) of MD simulation for the monomer form (Figure S1). The findings from these replicates indicate that the monomer form has a higher RMSD compared to the trimer spike protein (Figure 3a and Figure S1).

The root mean square fluctuations (RMSF) were computed on the Cα atoms of each residue from the spike protein, in order to trace their flexibility and thereby define the motions of different domains (Figure 3b). The RMSF findings in both forms (monomer and trimer) indicated that the amino acids in the RBD domain (residue range: 329–521) were highly fluctuating (Figure 1b and Figure 3b). In addition, the triplicate MD simulations of the monomer form, also suggests that the RBD domain has a higher RMSF in all three simulation replicates (Figure S1). These analyses correlate with previous studies [33,34,35,36,39]. Particularly, amino acids ranging from 470–490, responsible for interacting with the ACE2, that were highly fluctuating. Furthermore, examining other regions of the spike protein suggests that the S2 subunit domains (residue range: 850–1141; HR1, CH, and CD) showed the least fluctuations within the entire protein sequence (Figure 3b). This correlates with the cryo-EM studies performed on the spike protein; that the S2 subunit is more stable [18] compared to the RBD domain, and that this subunit is responsible for a highly stable postfusion conformation of the spike protein [18,32]. From the perspective of designing drugs, the more stable or less flexible a region is within a protein, the more accurately we can trace a better hit molecule. In the case of the spike protein, the RMSF findings guided us towards focusing on the S2 subunit (Figure 3b).

Moreover, by tracing the residues involved in the H-bond interactions between two monomers (i.e., chains A-B, A-C, or B-C) of the homotrimer, we observed that the RBD domain residues were also involved in intermolecular interactions with each other and with high occupancy (%). This suggests that intermolecular interactions between chains in the homotrimer might equilibrate the spike protein, and might stimulate conversion from a “down” to “up” conformation of the RBD domain that interacts with the ACE2 receptor (Figure S2 and Table S5).

The structural dynamics over the time course for spike protein in the monomer and the homotrimer form was monitored during MD simulations (Figure 3c,d). The monomeric spike protein in the solvent environment exhibited a movement from the “up” active state towards the “down” inactive state for the RBD domain (Figure 1c, Figure 3c, and Movie S1). These correlate with the previous findings that the RBD domain can form two different conformations, i.e., “down” and “up” states, which represents the host cell receptor-inaccessible and receptor-accessible, respectively [33,34,35,36]. The SARS-CoV-2 spike protein has a better binding affinity to the ACE2 receptor at two different “up” angles of the RBD domain compared to the SARS-CoV [40]. Figure 3c (and Movie S1) describes the conformational change in other regions of the spike protein, when the RBD domain moves towards an “up” to “down” state in the monomeric form.

Figure 3d (and Movie S2) represents the dynamics of the homotrimer spike protein, suggesting that the RBD domain of chain A opens more widely in its “up” state. Domains HR1, CH, and CD close to the viral transmembrane exhibited the least movement (Figure 3d) during MD simulations. In addition, exploration of the structural orientation of these S2 subunit domains (Figure 3d and Movie S2) suggests that they form a large pocket or cavity using three chains (or monomers) from a homotrimer spike protein. The slight movement observed in homotrimer during MD simulation of this cavity (Movies S1 and S2), and the structural orientation suggest that it could work as “bouncing spring” or “sarrus linkage”. One may postulate that, when the spike protein interacts with the ACE2 receptor, this “bouncing spring” or “sarrus linkage” movement may be important in the fusion of the virion with the host membrane. Additionally, this cavity from the spike protein could work as a platform for the design or development of new drug leads against this protein (Figure 3d). Such molecules might alter the trimer stability upon viral entry or upon viral coat assembly. There have been several studies performed to design drugs specific for the SARS-CoV-2 spike protein [20,38,39,40,42,44]; however, most of them are focused on the RBD domain. In addition, from our MD simulation and variability analysis (Figure 2 and Figure 3), the RBD domain is highly flexible and variable, therefore, drugging this variable site may be an obstacle in finding active hit molecules. By targeting the less variable S2 region, such as the cavity formed by the homotrimer (Figure 2 and Figure 3d) we suggest that this might be a novel approach to develop small molecule drug leads.

### 3.2. Potential Druggability of the Homotrimer Cavity

**Figure 4:**
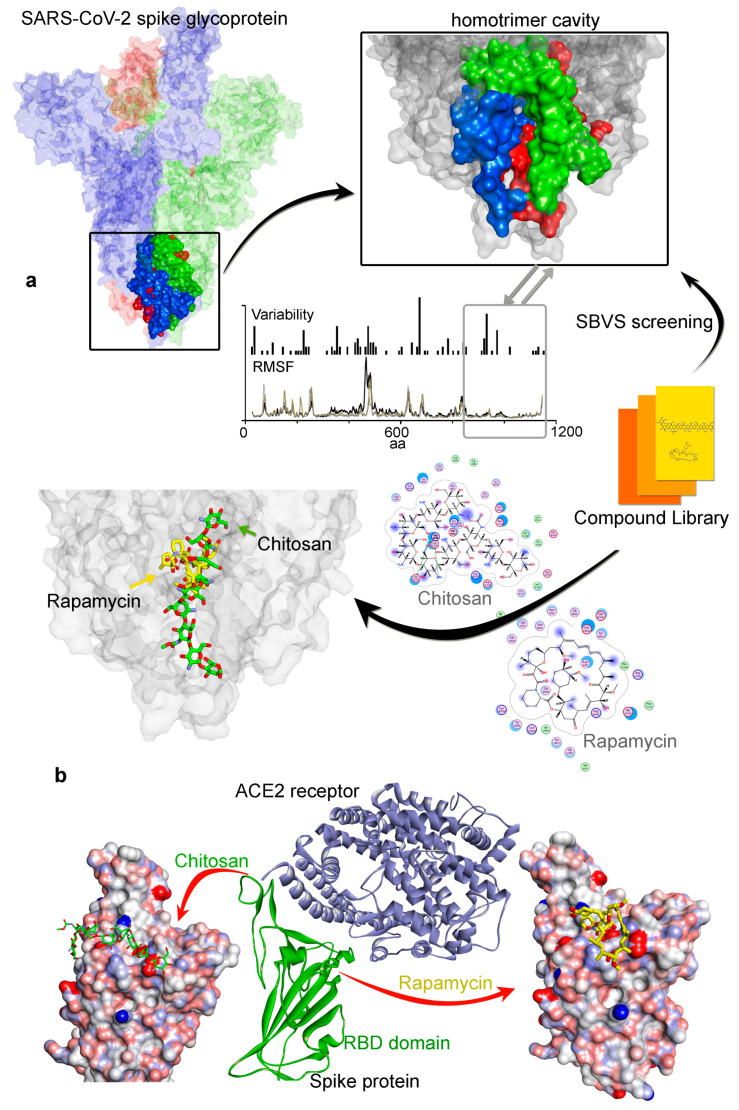
Targeting different pockets of the SARS-CoV-2 spike protein. (**a**) The homotrimer cavity from the SARS-CoV-2 spike glycoprotein bound to known compounds. (**b**) The ligands that were found interacting with the homotrimer cavity with high binding affinity were also docked with an interface formed by the spike proteins (RBD domain; PDB ID. 6lzg [90]) that interact with the ACE2 receptor. SBVS, structure-based virtual screening.

We next investigated the targetability of the trimer cavity formed by the S2 subunit (HR1, CH, and CD domains) in the spike protein (Figure 2 and Figure 3) using the MOE (Chemical Computing Group Inc.) package [74,75], before using it for high-throughput virtual screening (or SBVS) using a library of FDA approved drugs. The “Alpha Shapes” construction [75,77] geometric method was used to compute the possible residues that can be considered for ligand docking from this trimer cavity in the spike protein (Figure 4a).

High-throughput virtual screening is a powerful computational approach that is increasingly being used in the drug discovery process, through the in silico identification of novel hits from large compound databases [78]. We applied the SBVS approach to dock the molecules to the trimer cavity and to check its feasibility as a target. Ligand binding to this cavity might reduce or increase the “bouncing spring” movement in the spike protein, as observed in MD simulations (Figure 3, Movies S1 and S2). This perturbation might affect its interactions with the host cell receptor or the hinge movement of the RBD domain. The compounds that exhibit a relatively high binding affinity towards the SARS-CoV-2 spike glycoprotein trimer cavity with a binding affinity −35 to −71 kcal/mol (GBVI/WSA dG) were recorded. From the list of ligands showing best binding, the compounds that were already validated or suggested to be/can be active against the SARS-CoV-2 virus includes: Chitosan [54,55,56], Rapamycin [47,48,49], Everolimus (RAD001) [49], Paclitaxel [79], Ritonavir [48,50,51,52], SelaMeerin (Selamectin) [80], and Danoprevir [52] (Table 1).

Among these molecules Rapamycin and Everolimus drugs were previously identified as mTOR pathway inhibitors [47,50,81,82,83]. The antibacterial or antiparasitic drugs from the list are Chitosan [84] or SelaMeerin (Selamectin) [85], respectively. Paclitaxel, has been found to be previously target Bcl-2 and microtubule associated functions [86,87]. In addition, the FDA approved drugs that target the protease are: Ritonavir [88] and Danoprevir (ITMN-191) [89].

By docking known drugs within the trimer cavity of spike protein, the relative selectivity of the cavity suggests that the majority of higher-affinity drugs will have a molecular weight (MW) ≥ ~700 g/mol (Table 1 and Figure S3). However, this is with the certainty that compounds with high MW can form more interactions with the spike protein, in addition, our finding highlights the possibility that the trimer cavity can occupy large ligands deep inside the binding pocket (Figure 4a). Particularly, a specific class of ligands (mostly macrolide type) were found to exhibit a better fit to the trimer cavity (Figure 4a); for example, Rapamycin [47,48,49], Everolimus (RAD001) [49], Paclitaxel [79], and SelaMeerin (Selamectin) [80] (Figure 4a). The intermolecular interactions between the spike protein and the compounds suggest that residues from all three monomers (chains A, B, and C) are actively involved in binding to the drugs. In addition, placement of the compounds inside the trimer cavity suggests that they make use of the pocket space (forming different conformation) to form stable interactions with the spike protein (Figure 4a).

In order to check the selectivity of these ligands to the trimer cavity, we docked this same subset (Table 1) with the RBD domain of the viral spike protein (Figure 4b; PDB ID. 6lzg [90]). The RBD domain is involved in interacting with the ACE2 host cell receptor [16,26,30,38,39,40,41,42,90,91]. The docking suggests that all compounds from Table 1 have better binding affinity to the trimer cavity compared to that of the RBD domain. In addition, Chitosan [54,55,56,84] (a linear polysaccharide; −37.29 kcal/mol) could form a linear conformation in its structure when binding tothe RBD domain (Figure 4b and Table 1), whilst the same ligand (due to its molecular structural nature) can form a slightly folded shape (as shown in 2d-diagram; Figure 4a) within the trimer cavity. By contrast, Everolimus [49] (a macrolide type) exhibits high affinity for the trimer pocket, and very little selectivity for the RBD domain (Table 1). Moreover, ligands (Figure 4b) that interact with the RBD domain overlap with the region bound by CR3022 (a neutralizing antibody isolated from a convalescent SARS patient that, interacts with the receptor binding domain of the SARS-CoV-2 spike protein [92]).

## 4. Discussion

The SARS-CoV-2 virus causing COVID-19 disease uses the fusion spike glycoprotein to penetrate into the host cell, and therefore a detailed understanding of this protein forms a critical intervention point in the viral life-cycle. We interrogated the spike protein with a diversity of computational approaches. First, the variability in spike protein from 791 different viral genome sequences was evaluated. Residues in the S2 subunit (residue range: 816–1141; HR1, CH, and CD domains) were found to be less evolutionarily variable compared to other regions or domains. By contrast, residues H49Y, Q239K, V367F, V483A, S943P, K986P, and V987P were found to be the most common amino acid substitutions in the spike protein from related viruses. Secondly, MD simulations revealed that residues in RBD domain (residue range: 329–521) were more flexible compared to residues in the S2 subunit, making it more complicated for drug design strategies. An examination of less variable regions revealed that the HR1, CH, and CD domains (S2 subunit) located close to the viral transmembrane formed a large cavity or pocket that is formed from three spike monomers. The MD simulation traced an “up” active state and a “down” inactive state of the spike protein in its monomer form. Slight movement of the trimer cavity within this structural orientation suggests that it could work as “bouncing spring” or “sarrus linkage” when interacting with the host cell receptor.

The conversion between “up” and “down” states in the monomer form of spike protein using the in silico methods is defined by the MD field to be relatively fast. Nevertheless, there are different structural isoforms that have been identified on the spike protein using different experimental methods or virus strains. This indicates that, although conversion may be quick, there are structural endpoints which are “stable”. Using the recent cryo-EM structure of the SARS-CoV-2 spike protein [18]; an asymmetric hinge-like movement was observed in only one of the three RBD domains in the S1 subunit, which was also observed in MERS-CoV and SARS-CoV [33,34]. However, there are also other structures where all three RBD domains are in the “up” or “down” conformation [18,31,33,34,40]. These data suggest a physiological relevance due to heterogeneous protein conformational dynamics. For example, asymmetric conformational flexibility might have a functional role, perhaps in evading the exposure of B-cell epitopes (only one RBD domain is in the “up” conformation) and/or optimized interaction with the ACE2 receptor depending on virus strain. In addition, because of the “bouncing spring” mechanism (communication between the trimer pocket and the RBD domain conformation), it is possible that these different spike protein conformational isoforms provide another avenue to develop drug discovery programs that exploit and/or circumvent these dynamics.

Our investigation into the genomic variation within virus strains, as well as our findings from the MD simulations, identified a conserved trimer cavity or pocket formed by the S2 subunit in the spike protein. These findings suggest that a novel target, “the trimer cavity formed by spike protein oligomerization”, may be suitable to manipulate viruses of this class. Targeting the trimer pocket might identify a new functional class of drugs against this protein. Applying the SBVS approach, we docked drug libraries against the trimer cavity with the hypothesis that such a ligand might perturb the predicted “bouncing spring” movement and the homotrimer formation. Protein–ligand docking identified severalhits that have already been published or proposed to inhibit the SARS-CoV-2 virus in cell systems. For example, our studies suggest an action mechanism for molecules such as Chitosan and macrolide types (e.g., Rapamycin).

Based on the sequence variability of the coronavirus, including our findings from MD simulations of the spike protein, a conserved trimer cavity (HR1, CH, and CD domains) is a feature of the spike protein in most coronaviruses. Consistent with this, previous work has shown that the molecule EK1 exhibited potent inhibitory activity against all human coronaviruses (hCoVs) tested through binding to the C-terminal HR1 domain [37]. Additionally, the “up” and “down” conformations of RBD domain observed during MD simulations, supports that concept that the spike protein can also be a target of a possible IgG therapeutic [92]. From the list of the top compounds identified that dock into the trimer cavity, some of them have already been validated or suggested as SARS-CoV-2 virus inhibitors in cells, including; a Chitosan derivative [54,55,56], Rapamycin [47,48,49], Everolimus (RAD001) [49], Paclitaxel [79], Ritonavir [48,50,51,52], SelaMeerin (Selamectin) [80], and Danoprevir [52]. Among these, a modified polymeric version of the Chitosan drug (a top hit in our analysis) was recently shown to inhibit CoV replication with evidence that the molecule inhibits the binding of the viral spike protein to the host ACE2 receptor [54,55,56]. The protein–protein interaction map or the network-based methodologies [14,43] suggest that Sirolimus (Rapamycin) emerges as a common potential drug lead for repurposing against COVID-19. This Rapamycin (mTOR inhibitor) drug was found previously to disrupt LARP1 (La-related protein 1) and mTORC1 (mammalian target of rapamycin complex 1) binding, and has been shown to reduce MERS infection by ~60% in vitro [47]. The postulated geroprotectors, such as Sirolimus (Rapamycin) and its close derivative, the rapalog Everolimus (RAD001), decreased infection rates in a small sample of elderly patients [49].

Moreover, the drugs Sirolimus (Rapamycin) and Ritonavir are currently in clinical trials for repurposing against COVID-19 [48,50,51]. Sirolimus (Rapamycin) is registered in a clinical trial (NCT03901001 not yet recruiting) designed to evaluate adjunctive use of Sirolimus (Rapamycin) and Oseltamivir in patients hospitalized with influenza [47,48]. Ritonavir, a HIV protease inhibitor is in an open-label trial in hospitalized adults with severe COVID-19 [48,51]. The data from this small-sample clinical study showed that Danoprevir boosted by Ritonavir is safe and well tolerated in all patients [52]. Selamectin is a potential drug for treating COVID-2019 found active against the pangolin coronavirus GX_P2V, a workable model for SARS-CoV-2 research [80]. The antitumor drug Paclitaxel increases cellular methylglyoxal to virucidal levels, providing a rationale for repurposing Doxorubicin and Paclitaxel for COVID-19 treatment [79]. Nevertheless, whether the hit molecules we have identified that dock into the trimer cavity and impact on the virus life cycle requires orthogonal validation. We hope the findings of our study can help to understand the function of the highly conserved spike protein trimer cavity in the SARS-CoV-2 viral life cycle, as well as provide a novel approach to target this class of infectious disease by the examination of spike protein trimer stability and/or assembly.

## Figures and Tables

**Table 1:** The list of compounds showing the highest binding affinity to the trimer cavity from the spike protein, and the compounds that are already validated or suggested to be/can be active against the SARS-CoV-2 virus *.

Compounds Against SARS-CoV-2	GBVI/WSA dG (kcal/mol)		MW g/mol	Previous Target
	Trimer Cavity	RBD Domain		
Chitosan [54,55,56]	−67.49	−37.30	161.16 *	Antibacterial [84]
Rapamycin (Sirolimus) [47,48,49]	−49.28	−25.81	914.17	mTOR [47]
Paclitaxel [79]	−45.84	−32.42	853.92	Bcl-2, Microtubule Associated [86,87]
SelaMeerin (Selamectin) [80]	−44.24	−32.35	769.96	Antiparasitic [85]
Everolimus (RAD001) [49]	−41.80	0.29	958.22	mTOR [83]
Ritonavir [48,50,51,52]	−37.92	−24.11	720.94	HIV Protease [88]
Danoprevir (ITMN-191) [52]	−35.09	−30.80	731.83	Proteasome, HCV, Protease [89]

* Drugs in this list are in need of further clinical validation. MW for the monomer Chitosan compound is 161.16 g/mol, and for the entire Chitosan molecule is 1526.46 g/mol. GBVI/WSA, Generalized-Born Volume Integral/Weighted Surface area; mTOR, mammalian target of Rapamycin; HIV, human immunodeficiency viruses; HCV, hepatitis C virus; RBD, receptor binding domain; MW, molecular weight.

## Supplementary Materials

The following are available online at https://www.mdpi.com/2077-0383/9/5/1473/s1, File S1. Molecular dynamics simulation methods; Table S1: Variability in the SARS-CoV-2 spike protein for the entire sequence. The amino acid substitutions in each position across 791 SARS-CoV-2 strains from the GISAID database, Table S2: The receptor binding domain variability of the SARS-CoV-2 S protein. The amino acid substitutions in each position across 791 SARS-CoV-2 strains from the GISAID database, Table S3: S2 subunit sequence variability (residue range: 816-1141; HR1, CH, and CD domains) in the SARS-CoV-2 S protein. The amino acid substitutions in each position across 791 SARS-CoV-2 strains from the GISAID database, Table S4:The most common amino acid substitutions, position in the RBD domain and S2 subunit (HR1, CH, and CD domains), with comparison to the consensus sequence obtained from the alignment, Table S5: The hydrogen bond (H-bond) interactions between three monomers (i.e., chains A-B, A-C, and B-C) of the homotrimer spike protein traced during the MD simulations. In this table, the interaction pairs with donor or acceptor are mentioned, and residues showing occupancy (%) ≥10 % are considered; Figure S1: Triplicates of the MD simulations (or MD repeated three times) for the monomeric form of the SARS-CoV-2 spike protein. The plots represent the RMSF and RMSD obtained for the monomeric spike protein. In particular, the results of the monomeric form labeled as “0a” in the plots, is compared with the homotrimer spike protein in Figure 3, Figure S2: The intermolecular H-bond (hydrogen bond) interactions formed between three monomers (i.e., chains A-B, A-C, and B-C) of the homotrimer spike protein traced during the MD simulations, Figure S3: Correlation between the binding affinity (GBVI/WSA; kcal/mol) and molecular weight (MW; g/mol) of the compounds showing good or the best binding with the trimer cavity of the SARS-CoV-2 spike protein; Movie S1: The conformation dynamics of the monomeric form of the spike protein. This movie is generated using Chimera, taking into consideration the spike protein coordinates from 1 ns and 100 ns of the MD simulation, Movie S2: The conformation dynamics of the homotrimer spike protein observed during the MD simulations, focusing on the homotrimer cavity. The movie is generated using Chimera, taking into consideration spike protein coordinates from 1 ns and the average structure generated from the entire MD simulation (1–100 ns).
