# Supplementary material for: Highly Conserved Homotrimer Cavity Formed by the SARS-CoV-2 Spike Glycoprotein: A Novel Binding Site
Source: J Clin Med. 2020 May 14;9(5):1473. doi: 10.3390/jcm9051473 (PMC7290299; doi:10.3390/jcm9051473)
Supplement: Supplementary file 1 [file jcm-09-01473-s001.zip › jcm-796783-supplementary-for conversion/jcm-796783-supplementary-for conversion.docx]

**File S1. Molecular dynamics simulation methods.**

Inserted missing residues in the structure of the SARS-CoV-2 spike (S) glycoprotein (e.g., monomer chain A), as per Figure 1 in the main text include: 67AIHVSGTNGTKR78, 96EKS98, 143VYYHKNNKSWMES155, 177MDLEGKQGNF186, 247SYLTPGDSSSGWTA260, 329FPNITN334, 444KVGGN448, 455LFRKSNLKPFERDISTEIYQAGSTPCNGVEGFNCYF490, 501NG502, 621PVAIHADQLTPTWRVYSTG639, 673SYQTQTNSPGSASS686, 812PSK814, and 829ADAGFIKQYGDCLGDIAARDLICA852.

The molecular dynamics simulations (MDS) were performed using GROMACS 4.6.5 [1,2] and applying the CHARMM27 forcefield. For both monomer and homotrimer systems of the S protein, the PBC (periodic boundary conditions) were applied in all directions, and the simulation box was filled with water models applying simple point charge (SPC) parameters. In addition, to neutralize the simulation box, the Na^+^Cl^−^counter ions were added. Subsequently, the energy minimization of the model systems was performed (20,000 steps) using the steepest-descent algorithm until the systems reached its minimum energy. These minimized systems were then equilibrated to adjust water molecules with Na^+^Cl^-^ counter ions for 1000 ps using a constant number of particles, pressure, and temperature (NPT; isobaric-isothermal ensemble). The particle mesh ewald (PME) method was used for long range electrostatics. Moreover, for the van der Waals and Coulomb interactions a cutoff of 10 Å was applied. In order to restrain the bond lengths between the heavy atom and nonpolar hydrogen atoms, the LINear Constraint Solver constraints were applied [3,4]. The velocity-rescaling thermostat [5] was used to preserve a constant temperature (300 K). Parrinello-Rahman barostat [6,7] was applied to maintain constant pressure at 1.0 bar for the simulated systems. On the equilibrated systems, using the leapfrog integrator, the production run was performed for 100 ns for the monomeric and trimeric forms of the S proteins. The trajectories obtained as an output from the MD simulations were analyzed using the modulesfrom GROMACS [1,2], BIOVIA Discovery Studio (Dassault Systemes, BIOVIA Corp., San Diego, CA, USA), and visual molecular dynamics (VMD) tools [8]. The Chimera [9] package was used to generate movies for the coordinates of the S protein (monomer and homotrimer) obtained from the MD simulations.


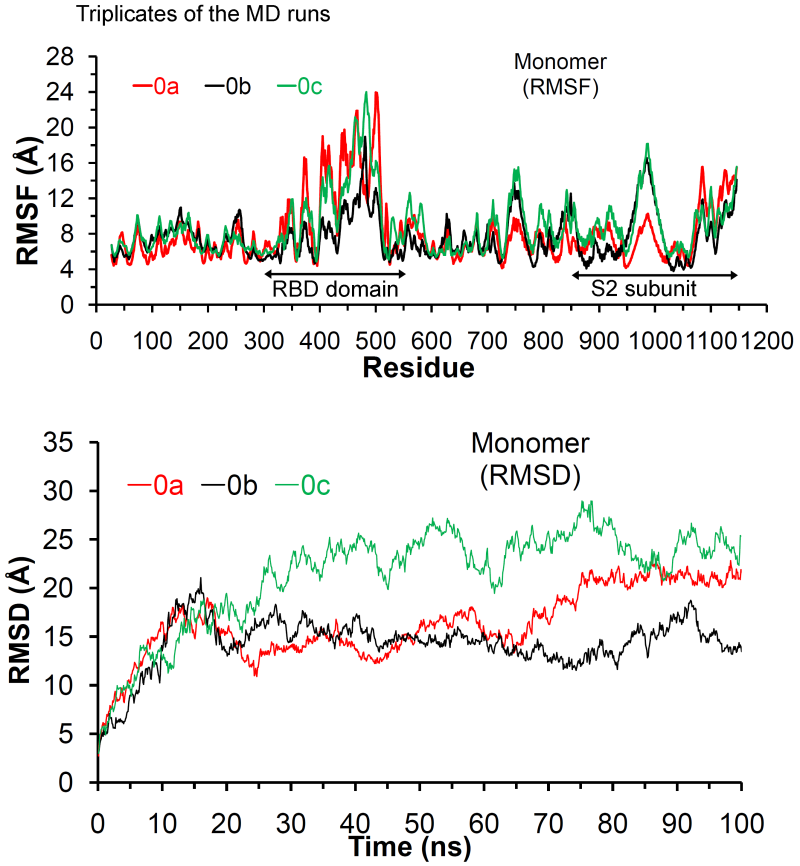


**Figure S1.** Triplicates of the MD simulations (or MD repeated three times) for the monomeric form of the SARS-CoV-2 spike protein. The plots represent the RMSF and RMSD obtained for the monomeric spike protein. In particular, the results of the monomeric form labeled as “0a” in the plots, is compared with the homotrimer spike protein in Figure 3. MD, molecular dynamics; SARS-CoV-2, severe acute respiratory syndrome coronavirus 2; RMSF, root mean square fluctuations; RMSD, root mean square deviation; RBD, receptor binding domain.

Supporting analysis for the figures from the main text

**Figure 2 in the main text.** The most common amino acid substitutions in the RBD domain and S2 subunit compared to the consensus sequence includes: H49Y Q239K, V367F, V483A, S943P, K986P, and V987P (Figure 2 and Table S1–S4).

Following previous studies that have explored sequence variability in the SARS-CoV-2 spike protein [10], it was confirmed that some of the first reported substitutions are high variability regions and common mutations. The phenylalanine substitution in position 32 exhibited a change not only to isoleucine (F32I), but also to serine (F32S). Other substitutions previously detected like H49Y, S247R, and V367F increase as a function of the total number of clinical isolates detected. The D614G substitution is of special interest as it has been detected in 30% of the GISAID strains (261 cases of the 791 total), consolidating this gene mutation that was previously reported once among 61 clinical isolates [10].

**Table S1.** (Attached as an excel file) Variability in the SARS-CoV-2 spike protein for the entire sequence. The amino acid substitutions in each position across 791 SARS-CoV-2 strains from the GISAID database. SARS-CoV-2, severe acute respiratory syndrome coronavirus 2; GISAID, Global Initiative on Sharing All Influenza Data platform.

**Table S2.** (Attached as an excel file) The receptor binding domain variability of the SARS-CoV-2 S protein. The amino acid substitutions in each position across 791 SARS-CoV-2 strains from the GISAID database. RBD, receptor binding domain.

**Table S3.** (Attached as an excel file) S2 subunit sequence variability (residue range: 816–1141; HR1, CH, and CD domains) in the SARS-CoV-2 S protein. The amino acid substitutions in each position across 791 SARS-CoV-2 strains from the GISAID database. HR1, heptad repeat 1; CH, central helix; CD, connector domain.

**Table S4.** The most common amino acid substitutions, position in the RBD domain and S2 subunit (HR1, CH, and CD domains), with comparison to the consensus sequence obtained from the alignment.

| **Domain** | **Position** | **Amino Acid Change** | **Consensus** | **Times Detected** |
| --- | --- | --- | --- | --- |
| **N-Ter** | 49 | Tyr | His | 6 |
|  | 239 | Lys | Gln | 6 |
| **RBD** | 367 | Phe | Val | 6 |
|  | 483 | Ala | Val | 3 |
| **S2 subunit** | 943 | Pro | Ser | 6 |
|  | 986 | Pro | Lys | 3 |
|  | 987 | Pro | Val | 3 |

RBD, receptor binding domain; HR1, heptad repeat 1; CH, central helix; CD, connector domain.

**Figure 3 in the main text.** The findings from RMSDs for both systems (monomer and trimer) suggests that the homotrimer form of S protein is more stable compared to the monomer form, and the monomeric spike protein has higher RMSD values of ~10–25 Å compared to all three chains or monomers (Figure 1a) in the trimer (~3–5 Å; Figure 3a). The monomer form of the spike protein exhibited stable RMSDs after 75 ns, while the homotrimer form showed stability throughout the MD simulations. In particular for the homotrimer system, the monomer chain A obtained a higher RMSD of about ~2 Å compared to other two monomers or chains (chain B and C). This chain A from homotrimer is suggested to be in “up” conformation [PDB ID: 6vsb) [10] that is involved in the interactions with the angiotensin converting enzyme-2 (ACE2) receptor.

**Intermolecular H-bond interactions between chains/monomers of the homotrimer.** The hydrogen bond (H-bond) interactions between three monomers (i.e., chains A-B, A-C, and B-C) of the homotrimer spike protein were traced during the MD simulations (Figure S2 and Table S5). These intermolecular interactions may facilitate the spike protein from the SARS-CoV-2 virus to obtain“down” to “up”conformation of the RBD domain that can bind with the angiotensin converting enzyme-2 (ACE2) receptor. The H-bonds were computed keeping the donor–acceptor distance cutoff ≤3.5 Å and donor-H-acceptor angle cutoff ≥ 160°−180°. The H-bond plots (Figure S2) explain that the pair chain A-chain C have a higher number (~25 H-bonds) of intermolecular interactions compared to the other two chain pairs (~15–20 H-bonds; chain A-chain B and chain B-chain C). Tracing residues involved in the interactions between chain A and chain B of the homotrimer, suggest that the RBD domain residues are also involved in the interactions with each other, and with high occupancy (%) (Table S5).


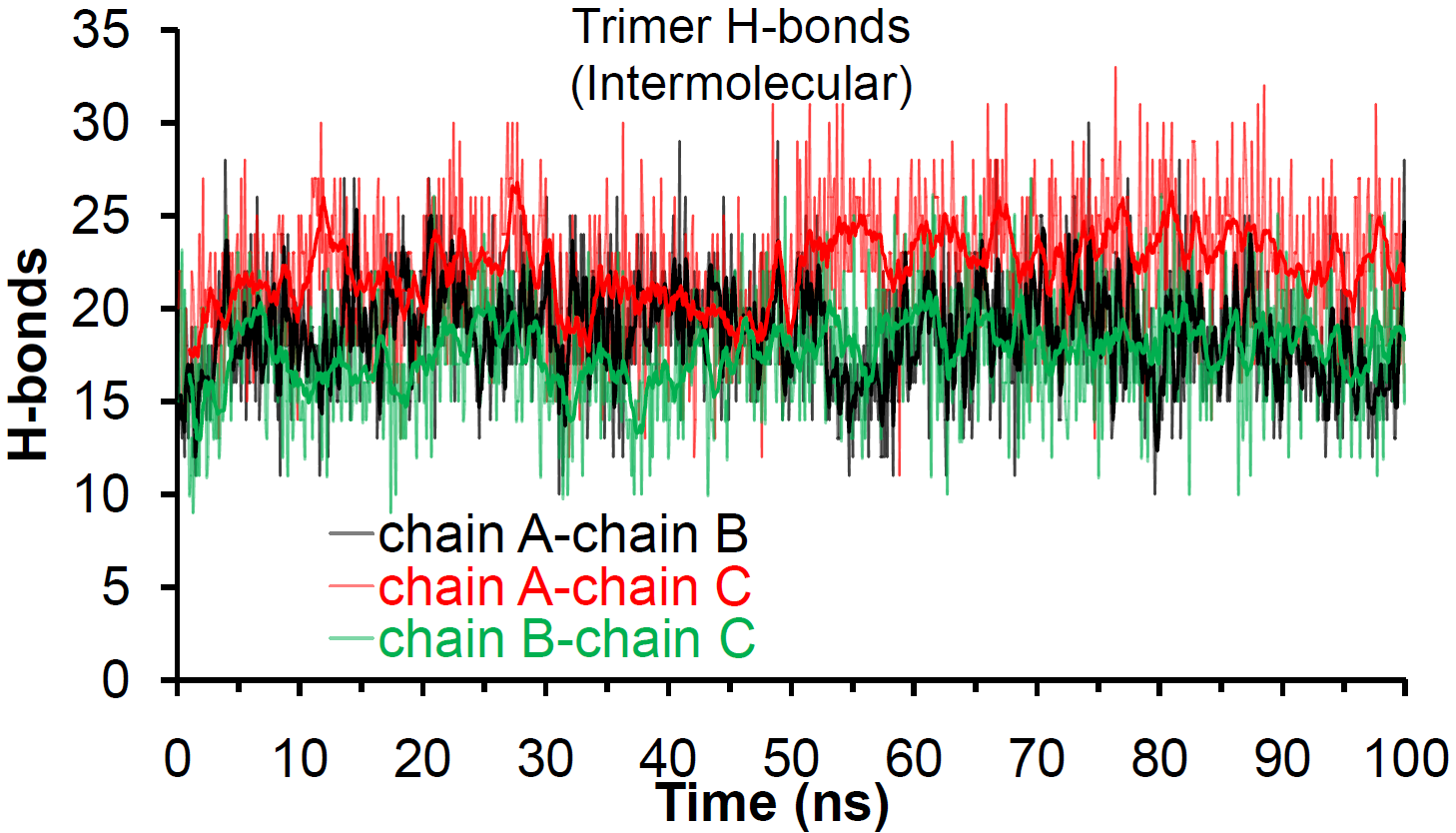


**Figure S2.** The intermolecular H-bond (hydrogen bond) interactions formed between three monomers (i.e., chains A-B, A-C, and B-C) of the homotrimer spike protein traced during the MD simulations.

**Table S5.** The hydrogen bond (H-bond) interactions between three monomers (i.e., chains A-B, A-C, and B-C) of the homotrimer spike protein traced during the MD simulations. In this table, the interaction pairs with donor or acceptor are mentioned, and residues showing occupancy (%) ≥10% are considered.

| **Chain A-Chain B** | | | **Chain A-Chain C** | | | **Chain B-Chain C** | | |
| --- | --- | --- | --- | --- | --- | --- | --- | --- |
| **Donor** | **Acceptor** | **Occup%** | **Donor** | **Acceptor** | **Occup%** | **Donor** | **Acceptor** | **Occup%** |
| Arg319 | Asp737 | 93.81 | Arg319 | Asp737 | 88.92 | Arg995 | Asp994 | 73.75 |
| Arg319 | Asp745 | 89.22 | Phe43 | Phe565 | 76.55 | Phe43 | Phe565 | 68.36 |
| Phe43 | Phe565 | 74.85 | Phe898 | Tyr707 | 70.66 | Phe898 | Tyr707 | 64.67 |
| Arg995 | Asp994 | 70.66 | Tyr200 | Glu516 | 69.66 | Ser383 | Asp985 | 58.78 |
| Thr547 | Asp979 | 64.37 | Arg1091 | Asp1118 | 66.77 | Arg319 | Asp745 | 57.29 |
| Phe898 | Tyr707 | 61.68 | Arg319 | Asp745 | 66.37 | Ala668 | Pro863 | 56.89 |
| Arg1039 | Glu1031 | 59.78 | Arg995 | Asp994 | 62.67 | Lys790 | Asn703 | 52.50 |
| Ala668 | Pro863 | 58.38 | Arg646 | Asp848 | 60.58 | Arg646 | Asp839 | 49.10 |
| Ser967 | Ala570 | 55.99 | Arg847 | Glu619 | 59.68 | Ile788 | Ala701 | 48.90 |
| Ser758 | Gln965 | 51.10 | Ser967 | Ala570 | 55.29 | Asn703 | Ile788 | 48.10 |
| Asn703 | Ile788 | 49.50 | Lys964 | Asp571 | 54.09 | Lys854 | Asp614 | 45.81 |
| Ile788 | Ala701 | 49.10 | Arg1039 | Glu1031 | 52.50 | Arg1039 | Glu1031 | 44.11 |
| Arg646 | Asp848 | 48.90 | Ala713 | Gln895 | 52.20 | Arg646 | Glu868 | 43.41 |
| Lys854 | Asp614 | 48.60 | Ser383 | Asp985 | 51.70 | Arg567 | Phe43 | 37.03 |
| Ser968 | Tyr756 | 44.51 | Thr859 | Asp614 | 49.80 | Lys790 | Glu702 | 36.33 |
| Gln564 | Lys41 | 44.41 | Lys790 | Asn703 | 49.00 | Arg847 | Asp614 | 34.13 |
| Lys790 | Asn703 | 41.72 | Ser968 | Tyr756 | 48.60 | Arg1091 | Asp1118 | 31.54 |
| Arg1091 | Asp1118 | 40.52 | Arg567 | Phe43 | 47.50 | Gln1005 | Gln1002 | 30.34 |
| Lys790 | Glu702 | 40.12 | Gln564 | Lys41 | 45.71 | Arg408 | Asp405 | 28.94 |
| Arg1019 | Glu1017 | 38.32 | Lys854 | Asp614 | 45.21 | Ala713 | Gln895 | 26.75 |
| Ala713 | Gln895 | 34.83 | Thr547 | Asp979 | 45.11 | Arg995 | Gln755 | 26.15 |
| Gln895 | Asn1074 | 34.23 | Lys790 | Glu702 | 42.71 | Ser967 | Asp571 | 25.25 |
| Tyr707 | Asp796 | 33.93 | Lys458 | Asp389 | 41.92 | Gln115 | Arg466 | 22.46 |
| Arg567 | Phe43 | 31.64 | Ala668 | Pro863 | 33.73 | Lys386 | Asp985 | 22.06 |
| Asn317 | Asp737 | 31.54 | Ser758 | Gln965 | 32.44 | Tyr200 | Glu516 | 21.86 |
| Arg357 | Glu169 | 28.54 | Asn703 | Ile788 | 29.84 | Asn234 | Glu465 | 21.26 |
| Lys964 | Asp571 | 28.34 | Asn709 | Asp796 | 29.74 | Lys854 | Phe592 | 20.86 |
| Gly669 | Leu864 | 27.84 | Ile788 | Ala701 | 27.74 | Tyr421 | Asn370 | 20.26 |
| Arg646 | Asp867 | 19.76 | Ala372 | Asn481 | 27.54 | Arg1091 | Glu1092 | 19.66 |
| Phe565 | Lys41 | 18.66 | Asn487 | Ser371 | 26.85 | Arg1019 | Glu1017 | 17.27 |
| Thr961 | Gln762 | 13.17 | Gly669 | Leu864 | 26.35 | Thr415 | Asp985 | 16.27 |
| Val705 | Lys790 | 11.78 | Tyr489 | Leu368 | 24.65 | Gln965 | Gln755 | 15.97 |
| Gln965 | Tyr756 | 11.68 | Ser383 | Arg983 | 24.25 | Val705 | Lys790 | 15.87 |
| Gln563 | Asp40 | 11.38 | Phe565 | Lys41 | 21.26 | Gln506 | Asn501 | 14.67 |
|  |  |  | Asn394 | Tyr200 | 20.46 | Ser968 | Gln755 | 14.57 |
|  |  |  | Gln895 | Ala706 | 17.96 | Asn616 | Asp843 | 14.27 |
|  |  |  | Lys386 | Asp985 | 15.47 | Tyr369 | Asp420 | 14.17 |
|  |  |  | Gln784 | Asp1041 | 15.07 | Lys113 | Glu471 | 13.37 |
|  |  |  | Lys202 | His519 | 12.97 | Gly669 | Leu864 | 11.98 |
|  |  |  | Thr478 | Asn370 | 11.98 | Gln965 | Ser758 | 10.58 |
|  |  |  | Gly593 | Asp737 | 11.18 |  |  |  |
|  |  |  | Lys386 | Leu984 | 10.98 |  |  |  |

MD, molecular dynamics.

**Figure 4 in the main text**. Findings from the genomics analysis of the virus, as well as from the MD simulations of the spike protein, indicate that a conserved region with significant mechanistic importance may be a trimer cavity or pocket formed by the S2 subunit (HR1, CH, and CD domains). Therefore, we investigated the targetability of this region or the trimer pocket using the MOE (Chemical Computing Group Inc.), before using it for high-throughput virtual screening (or SBVS) using a library of FDA approved drugs. The “Alpha Shapes” construction [11,12] geometric method was used to compute the possible residues that can be considered for ligand docking from this trimer cavity in the S protein (Figure 4a). This method classifies the alpha spheres as either “hydrophobic” or “hydrophilic (for lone pair active; LPA)” depending on whether the sphere is in a good hydrogen bonding spot in the receptor. The “AphaShapes” method identified the largest cluster or active sites residues from each monomer (chains A, B, and C) of the homotrimer spike protein were: W886, Y904, N907, G908, I909, G910, V911, T912, Q913, N914, E1031, G1035, Q1036, S1037, K1038, R1039, V1040, D1041, G1046, Y1047, H1048, K1086, H1088, P1090, R1091, E1092, G1093, V1094, Q1106, R1107, N1108, E1111, Q1113, T1117, D1118, N1119, T1120, F1121, V1122, S1123, G1124, D1139, P1140, L1141, and E1144 (Figure 4a).


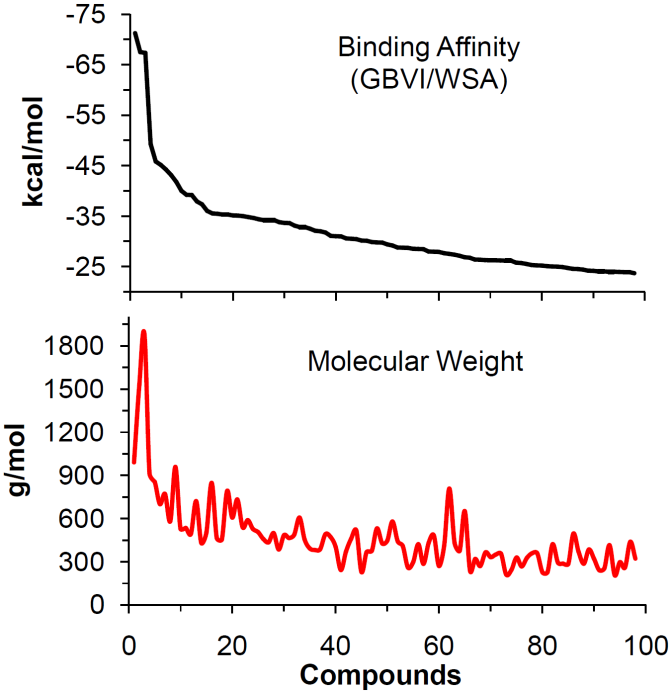


**Figure S3.** Correlation between the binding affinity (GBVI/WSA; kcal/mol) and molecular weight (MW; g/mol) of the compounds showing good or the best binding with the trimer cavity of the SARS-CoV-2 spike protein. GBVI/WSA, Generalized-Born Volume Integral/Weighted Surface area; MW, molecular weight.

Movie attached as separate files

**Movie S1.** The conformation dynamics of the monomeric form of the spike protein. This movie is generated using Chimera [9], taking into consideration the spike protein coordinates from 1 ns and 100 ns of the MD simulation.

**Movie S2.** The conformation dynamics of the homotrimer spike protein observed during the MD simulations, focusing on the homotrimer cavity. The movie is generated using Chimera [9], taking into consideration spike protein coordinates from 1 ns and the average structure generated from the entire MD simulation (1–100 ns).

References

1. Pronk, S.; Páll, S.; Schulz, R.; Larsson, P.; Bjelkmar, P.; Apostolov, R.; Shirts, M.R.; Smith, J.C.; Kasson, P.M.; Van der Spoel, D.; et al. GROMACS 4.5: A high-throughput and highly parallel open source molecular simulation toolkit. *Bioinformatics* **2013**, *29*, 845–854.
2. Berendsen, H.; Spoel, D.V.D.; Drunen, R.V. GROMACS: A message-passing parallel molecular dynamics implementation. *Comput. Phys. Commun.* **1995**, *91*, 43–56.
3. Darden, T.; York, D.; Pedersen, L. Particle mesh Ewald: AnN⋅log(N) method for Ewald sums in large systems. *J. Chem. Phys.* **1993**, *98*, 10089–10092.
4. Hess, B.; Bekker, H.; Berendsen, H.J.C.; Fraaije, J.G.E.M. LINCS: A linear constraint solver for molecular simulations. *J. Comput. Chem.* **1997**, *18*, 1463–1472.
5. Bussi, G.; Donadio, D.; Parrinello, M. Canonical sampling through velocity rescaling. *J. Chem. Phys.* **2007**, *126*, 014101.
6. Parrinello, M.; Rahman, A. Polymorphic transitions in single crystals: A new molecular dynamics method. *J. Appl. Phys.* **1981**, *52*, 7182–7190.
7. Gunsteren, W.F.V.; Berendsen, H.J.C. A Leap-frog algorithm for stochastic dynamics. *Mol. Simulat.* **1988**, *1*, 173–185.
8. Humphrey, W.; Dalke, A.; Schulten, K. VMD: Visual molecular dynamics. *J. Mol. Graph.* **1996**, *14*, 33–38.
9. Pettersen, E.F.; Goddard, T.D.; Huang, C.C.; Couch, G.S.; Greenblatt, D.M.; Meng, E.C.; Ferrin, T.E. UCSF Chimera--A visualization system for exploratory research and analysis. *J. Comput. Chem.* **2004**, *25*, 1605–1612.
10. Wrapp, D.; Wang, N.; Corbett, K.S.; Goldsmith, J.A.; Hsieh, C.L.; Abiona, O.; Graham, B.S.; McLellan, J.S. Cryo-EM structure of the 2019-nCoV spike in the prefusion conformation. *Science* **2020**, *367*, 1260–1263.
11. Molecular Operating Environment. Available online: https://www.chemcomp.com/Products.htm (accessed on 22 March 2020).
12. Edelsbrunner, H. The union of balls and its dual shape. *Discrete Comput. Geom.* **1995**, *13*, 415–440.
